# Mitophagy in carcinogenesis and cancer treatment

**DOI:** 10.1007/s12672-021-00454-1

**Published:** 2021-12-01

**Authors:** Tatiana V. Denisenko, Vladimir Gogvadze, Boris Zhivotovsky

**Affiliations:** 1 MV Lomonosov Moscow State University, 119991 Moscow, Russia; 2 Institute of Environmental Medicine, Division of Toxicology, Karolinska Institutet, Box 210, 171 77 Stockholm, Sweden

**Keywords:** Mitophagy, Autophagy, Cancer, Homeostasis

## Abstract

In order to maintain a functional mitochondrial network, cells have developed a quality control mechanism, namely mitophagy. This process can be induced through different pathways. The most studied is the so-called PINK1/Parkin pathway, which is associated with ubiquitylation of several mitochondrial proteins that were initially found to be related to Parkinson’s disease. Another type of mitophagy is known as receptor-mediated mitophagy, which includes proteins, such as BNIP3 and BNIP3L, also known as Nix. Through these two mechanisms, mitophagy fulfills its functions and maintains cellular homeostasis. Here, we summarize the current knowledge about the mechanisms of mitophagy regulation and their interplay with cancer progression as well as anticancer treatment.

## Introduction

Mitochondria are “the powerhouse of the cell” responsible for ATP synthesis via oxidative phosphorylation. Apart from energy production, mitochondria are involved in various intracellular processes, including ROS production, regulation of ion homeostasis, adaptation to stresses, initiation of cell death, etc. Dysfunctional mitochondria have a reduced capacity to carry out oxidative phosphorylation (OXPHOS); moreover, they produce more reactive oxygen species (ROS), which can cause cell damage [1]. Uncontrolled mitochondrial oxidative stress may contribute to different pathological states and diseases, including cancer [2].

Therefore, in order to maintain a functional mitochondrial network, cells have developed a quality control mechanism, namely mitophagy [3]. Dysregulation of mitophagy is frequently associated with different pathological situations including cancer. Here, we try to summarize the current knowledge about the main mechanisms of mitophagy regulation and their interplay with cancer progression. Furthermore, we highlight novel approaches for cancer treatment associated with mitophagy.

## Molecular mechanisms of mitophagy

Macroautophagy (hereafter referred to as autophagy) is a highly conserved pathway that captures and degrades proteins, as well as cellular organelles, in order to sustain cell survival during starvation and other stress situations [4]. Autophagy is initiated by the formation of double-membrane structures, called “autophagosomes”, that enclose cellular cytoplasmic constituents and, subsequently, fuse with lysosomes to form autophagolysosomal structures to degrade the content by lysosomal hydrolysis [4].

Mitophagy is a selective form of autophagy allowing for the degradation of damaged or dysfunctional mitochondria. Dysfunctional mitochondria are unable to carry out OXPHOS properly due to mitochondrial membrane depolarization and further accumulation of ROS, resulting in a significant increase in overall cellular oxidative stress [5]. Since mitochondria form a highly dynamic network, the dysfunctional mitochondrion needs to be isolated from the healthy network; this process requires the precise coordination of mitochondrial dynamics [6]. Mitochondrial dynamics i.e., fission (fragmentation) and fusion (elongation), defines mitochondrial shape, quality and quantity and regulates different cellular functions including proliferation, migration and metabolism [3, 7, 8]. Mechanistically, fission and fusion are tightly regulated by guanosine triphosphatases (GTPases) [9]. Mitochondrial fission is driven by dynamin-related protein 1 (Drp1) [10], a GTP-binding protein that can be recruited to the mitochondrial membrane and, with the assistance of adaptor proteins like Fis1, MFF, MID49 and MID51, forms a ring structure around the mitochondrion, thereby inducing the division of the mitochondrial membrane [10–12]. The fission of mitochondria is regulated by the phosphorylation status of Drp1 [13]. Thus, the phosphorylation of Drp1 at Ser585 by CDK 1/Cyclin B activates mitochondrial fission in mitotic cells, whereas phosphorylation of Drp1 at Ser637 leads to the inhibition of fission [14]. Mitochondrial fusion is regulated by mitofusins 1 and 2 (Mfn1, 2) at the outer mitochondrial membrane (OMM), whereas inner mitochondrial membrane (IMM) fusion is induced by the cristae-shaping protein Opa1 [10]. Mitochondrial fission can produce impaired daughter mitochondria that will be utilized by mitophagy [15].

**Fig. 1:**
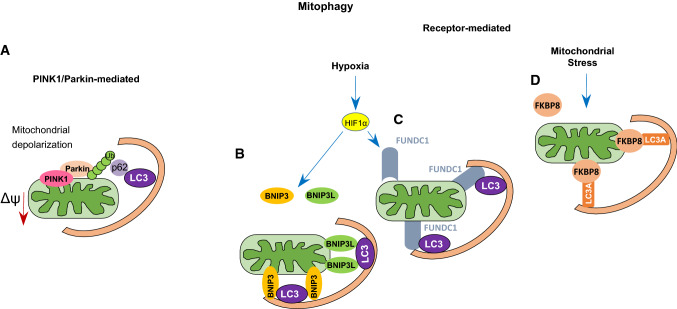
Schematic representation of PINK1/Parkin- and receptor-mediated mitophagy (see the text for the details). **A** PINK1/Parkin-mediated mitophagy, **B**–**D** BNIP3- FUNDC1- FKBP8-mediated mitophagy, respectively

Mitophagy can be induced through different pathways. The most studied is the so-called PINK1/Parkin pathway (Fig. 1A) [3]. It is associated with the ubiquitynation of several mitochondrial proteins that were initially found to be related to Parkinson’s disease [16]. PINK1 is a serine/threonine kinase that contains a mitochondrial targeting sequence at its N-terminus [17, 18]. Under physiological conditions, PINK1 is transferred into the IMM by translocase of the outer membrane (TOM) and translocase of the inner membrane (TIM) complexes, where it is cleaved by the mitochondrial protease PARL (presenilin-associated rhomboid-like), and further degraded by the proteasome. PINK1 transport into mitochondria is driven by the mitochondrial membrane potential. In depolarized mitochondria, PINK1 stays associated with the OMM, where it is activated through autophosphorylation and phosphorylates ubiquitin chains [19, 20]. In turn, phosphorylated ubiquitin chains facilitate the recruitment of Parkin, an E3 ubiquitin ligase, and amplification of mitophagy signal [3, 21], triggering the sequestration of impaired mitochondria [6].

Another type of mitophagy is known as receptor-mediated mitophagy (Fig. 1B–D). Different types of receptors have been reported to contribute to the elimination of mitochondria under physiological and pathological conditions, including BNIP3 and BNIP3L (also known as NIX), FUNDC1 and BCL2L13 [22–26]. BCL2/adenovirus E1B 19 kDa protein-interacting protein 3 (BNIP3) and BNIP3-like (BNIP3L/NIX) are important mediators of hypoxia-induced mitophagy [27, 28]. Indeed, both BNIP3 and NIX are transcriptional targets of HIF1 (hypoxia-inducible factor 1 alpha) (Fig. 1) [6, 29]. However, BNIP3 and NIX can also be regulated by other transcription factors like FOXOa3 or NF-κB, implying their involvement in signaling pathways beyond hypoxia [30, 31]. BNIP3 and Nix contain LIR (LC3-interacting region) domains, which provide direct binding to microtubule-associated proteins 1A/1B light chain 3B (MAP1LC3B) to induce mitophagy [29]. The interaction of BNIP3 with MAP1LC3B requires phosphorylation of its serine residues Ser17 and Ser24 [6]. Both BNIP3 and NIX belong to the pro-apoptotic Bcl-2 family of proteins. Expression of both has been linked to non-apoptotic cell death in response to various stresses, and both proteins are categorized as BH3-only proteins [2, 27]. However, further studies have demonstrated that the BH3 domain in BNIP3 and NIX is atypical, leading to the reduced pro-apoptotic function of both proteins [32, 33].

Mitophagy induced by BNIP3 is associated with mitochondrial fragmentation and perinuclear clustering [2, 34, 35]. Thus, BNIP3 overexpression activates mitochondrial fragmentation through inhibition of the fusion protein Opa1, leading to cristae remodeling [36, 37]. Additionally, BNIP3-induced mitophagy stimulates the translocation of the fission protein Drp1 to mitochondria, whereas the overexpression of either Mfn1 or dominant negative Drp1 inhibits BNIP3-dependent mitophagy [38]. Apparently, there is an interplay between BNIP3 and regulators of mitochondrial dynamics [2].

Another mitophagy receptor activated by hypoxia is FUNDC1 (FUN14 domain-containing protein 1) [39, 40]. In response to hypoxia, FUNDC1 binds to MAP1LC3B via its LIR motif and facilitates mitophagy through autophagosome formation [39]. Phosphorylation of FUNDC1 at a tyrosine residue in the LIR motif by SCR1 kinase inhibits mitophagy [39]. On the other hand, the phosphorylation of serine 17 by ULK-1 facilitates the FUNDC1-MAP1LC3B interaction, thereby accelerating mitophagy [39]. Thus, the FUNDC1 phosphorylation state determines its affinity for MAP1LC3B and activates mitophagy. The PGC-1α-NRF1 pathway is a crucial regulator of mitochondrial biogenesis. PGC-1α and NRF1, involved in the regulation of mitochondrial biogenesis, has been shown to upregulate the expression of FUNDC1, thereby stimulating mitophagy and promoting mitochondrial turnover [41].

Autophagy regulator Ambra1 was shown to induce mitochondrial depolarization and subsequent mitophagy activation via a Parkin-independent pathway. Ambra1 interacts with the E3 ubiquitin ligase HUWE1, which induces mitophagy through ubiquitylation and further degradation of mitofusin 2 (Mfn2) [42]. After mitophagy induction, Ambra1 binds to MAP1LC3B, leading to autophagosome formation [42]. In addition, Ambra1 may accelerate mitophagy through the PINK1/Parkin pathway [43].

FKBP prolyl isomerase 8 (FKBP8/FKBP38), another mitophagy receptor, belongs to the immunophilin family [44]. FKBP8 is normally localized to the OMM and recruits MAP1LC3A by its LIR motif [44]. Overexpression of FKBP8 stimulates mitochondrial fission, followed by mitophagy induction [44]. Unlike other mitophagy receptors, FKBP8 avoids degradation in the autophagosome during mitophagy and translocates to the endoplasmic reticulum, where it binds to Bcl-2 [44]. Thus, through these two mechanisms, mitophagy plays an important role in the mitochondrial stress response, also providing mitochondrial quality control and the maintenance of homeostasis.

## The role of mitophagy in tumorigenesis

Although the role of mitophagy in tumorigenesis remains incompletely understood, recent evidence demonstrates that the dysregulation of mitophagy is frequently associated with cancer [17]. Like autophagy, mitophagy plays a dual role in cancer and may either promote or suppress tumorigenesis, depending on the tumor type and molecular context [45]. Thus, the loss of function of several mitophagy-related genes results in the inhibition of mitophagy and further accumulation of dysfunctional mitochondria, thereby contributing to tumorigenesis. On the other hand, mitophagy may act as a tumor-promoting mechanism and thus contribute to cancer cell survival under stress conditions [3].

As mentioned above, the PINK1/Parkin pathway is one of the main pathways of mitophagy [46]. The loss of function of Parkin can suppress mitophagy and contribute to carcinogenesis [3]. For instance, Parkin/PARK2 gene mutations have been detected in lung and breast cancers as well as in glioma [47–49]. In these tumors, loss or partial deletion of PARK2 leads to the acceleration of tumor progression, demonstrating that PARK2 mutations may function as a driver mutation [50]. Additionally, amplification of PARK2 contributes to the inhibition of hepatocellular carcinoma and colon cancer cell growth [51]. PINK1 and Parkin have been shown to suppress HIF1α stabilization [52–54]; Parkin interacts directly with HIF-1α, promoting its ubiquitination at K477 and further degradation, which in turn suppresses breast cancer metastasis [54].

Parkin has also been reported to be a p53 target gene involved in the regulation of glucose metabolism [55]. Parkin downregulation results in a switch to aerobic glycolysis (Warburg effect), whereas restoration of Parkin expression in cancer cells reverses the Warburg effect [50]. These observations provide the evidence that Parkin acts as a tumor suppressor and its downregulation contributes to the progression of different types of tumors. However, Parkin knockout in a mouse model of melanoma suppressed tumor growth and metastatic dissemination through the downregulation of Parkin-related ubiquitination of Mfn2 [56, 57]. Furthermore, in vitro studies have confirmed that Parkin knockout significantly inhibits the growth and migration of melanoma cells, suggesting that Parkin may also contribute to tumor progression [56, 57].

The mitophagy adaptors BNIP3 and NIX, induced by hypoxia, have also been found to play important roles in tumorigenesis [58]. BNIP and NIX have been shown to act as tumor suppressors in different cancers [59]. For instance, in a mouse model of mammary tumors, BNIP3 deletion stimulated tumor growth linked with mitochondrial dysfunction, activation of HIF and elevated ROS production [59]. Furthermore, BNIP3 loss in this type of tumor was associated with increased angiogenesis, glycolytic shift, and metastatic dissemination [59].

Likewise, knockout of the NIX gene accelerates tumor growth, whereas upregulation of NIX expression induced by p53 contributes to tumor cell apoptosis [60]. Interestingly, in pancreatic cancer, BNIP3 downregulation contributes to chemotherapy resistance and is associated with poor patient prognosis, implying a tumor-suppressing role of BNIP3 [61]. However, NIX has been shown to promote pancreatic carcinogenesis, whereas loss of NIX results in the restoration of mitochondrial function and delays cancer progression [62]. The different roles of BNIP3 and NIX in pancreatic cancer require further investigation. Other reports have shown that increased expression of BNIP3 detected in hypoxic regions of lung and prostate cancers, glioblastoma multiforme, cervical tumors, endometrial cancer, breast carcinomas and gastric adenocarcinomas correlates with an aggressive tumor phenotype and a poor prognosis [58, 63–65]. Nix is also highly expressed in hypoxic tumor cells, and Nix-mediated mitophagy promotes cancer cell survival in glioblastoma and pancreatic cancers, associated with poor patient prognosis [66]. In addition, BNIP3-induced mitophagy promotes cell migration and metastasis, thereby contributing to different stages of the metastatic cascade, including cytoskeleton remodeling and invasion [67, 68]. Thus, the controversial roles of BNIP3 and NIX in cancer progression can be explained by different types of tumors and molecular contexts.

FUNDC1 is also involved in the regulation of cancer initiation and progression [69]. In cervical cancer, the expression of FUNDC1 is significantly upregulated in tumors as compared to normal tissues [70]. The overexpression of FUNDC1 is associated with tumor progression and poor patient prognosis, indicating a tumor-promoting role of FUNDC1 in cervical cancer [70]. FUNDC1 has also been reported to inhibit hepatocellular carcinoma (HCC) progression by suppressing activation of the inflammasome in mice [71]. The inflammasome is a molecular platform that promotes inflammatory cell death through the activation of caspase-1 and interleukin synthesis [72]. FUNDC1 depletion in hepatocytes results in the accumulation of damaged mitochondria and increases the inflammatory response, including caspase-1 activation and stimulation of JAK/STAT and NF-κB signaling, leading to the progression of HCC [71]. In this context, mitophagy plays important role in the regulation of inflammasome activation by preventing the accumulation of damaged mitochondria and tumor progression.

Altogether, these studies provide evidence that the role of mitophagy in cancer progression is more complex than was suggested by previous studies demonstrating both tumor promoting and tumor suppressing activities depending on tumor type, stage, or metabolic activity. The modulation of mitophagy may represent new attractive approach for cancer treatment.

## Why should mitochondria be deleted?

As stated above, mitophagy is an important quality control mechanism involved in the regulation of homeostasis and the maintenance of a healthy mitochondrial network [73]. Thus, when mitophagy is impaired, the accumulation of dysfunctional mitochondria occurs, which might affect cell homeostasis and lead to the occurrence of related diseases. For instance, mitochondrial dysfunction may contribute to the progression of various diseases, including cancer, through different mechanisms including increased ROS generation, metabolic reprogramming, and production of oncometabolites [45]. At the physiological level, the production of ROS is tightly regulated by antioxidant systems, whereas under pathological conditions, the ROS level is increased, leading to the oxidative stress and damage to mitochondrial proteins, lipids and DNA [74]. For instance, oxidative damage to lipids may cause lipid peroxidation and oxysterol formation, leading to the loss of membrane properties. Oxidative damage to proteins may endow proteins with oxidative modifications such as the oxidation of sulfur in methionine or cysteine, further leading to protein dysfunction [75]. ROS overproduction significantly contributes to carcinogenesis through the accumulation of DNA mutations [76]. Furthermore, ROS have been reported to activate different signaling pathways associated with cancer, including the PI3K pathway. Moreover, ROS promote the inactivation of the tumor suppressor PTEN (phosphatase and tensin homolog), the main target of the PI3K pathway, by oxidizing active-site cysteine residues [76, 77].

Dysregulation of mitophagy also affects the energy metabolism of the cell. Indeed, cancer cells frequently show a shift towards aerobic glycolysis for energy production, even under normoxic conditions, by reducing oxidative phosphorylation [2]. This shift could be explained by altered expression of key metabolic enzymes including pyruvate kinase M2 (PKM2), succinate dehydrogenase, phosphoglycerate dehydrogenase and isocitrate dehydrogenase in cancer cells. Parkin has been reported to suppress glycolysis through interactions with PKM2, promoting its ubiquitination and leading to the inhibition of its enzymatic activity [78]. Parkin has also been shown to mediate the ubiquitination and degradation of HIF1α, thereby preventing the activation of its transcriptional targets, including proteins involved in glycolysis [78]. Likewise, PINK depletion results in the Warburg effect through the stabilization of HIF1α and reduced activity of PKM2 [52]. Furthermore, BNIP3-dependent mitochondrial clearance has been reported to suppress the glycolytic shift in wild-type p53 radioresistant cells, whereas defects in mitophagy are associated with the accumulation of dysfunctional mitochondria, which contribute to the glycolytic phenotype in radioresistant cells of head and neck squamous cell carcinoma [17].

Mitophagy has also been reported to be involved in inflammasome activation [71]. Mitophagy plays an important role in suppressing inflammasome activation, thereby preventing mitochondrial damage and tumorigenesis. For instance, FUNDC1-activated mitophagy suppresses HCC development through the limitation of inflammasome activation in a mouse model. FUNDC1 knockdown in hepatocytes led to the accumulation of damaged mitochondria, elevated release of pro-inflammatory cytokines and inflammasome activation, contributing to carcinogenesis [71].

Thus, mitophagy dysfunction and the consequent accumulation of damaged mitochondria could contribute to carcinogenesis through different mechanisms, including increased ROS generation, inflammation, and cellular bioenergetics.

## Mitophagy and treatment of cancer

**Fig. 2:**
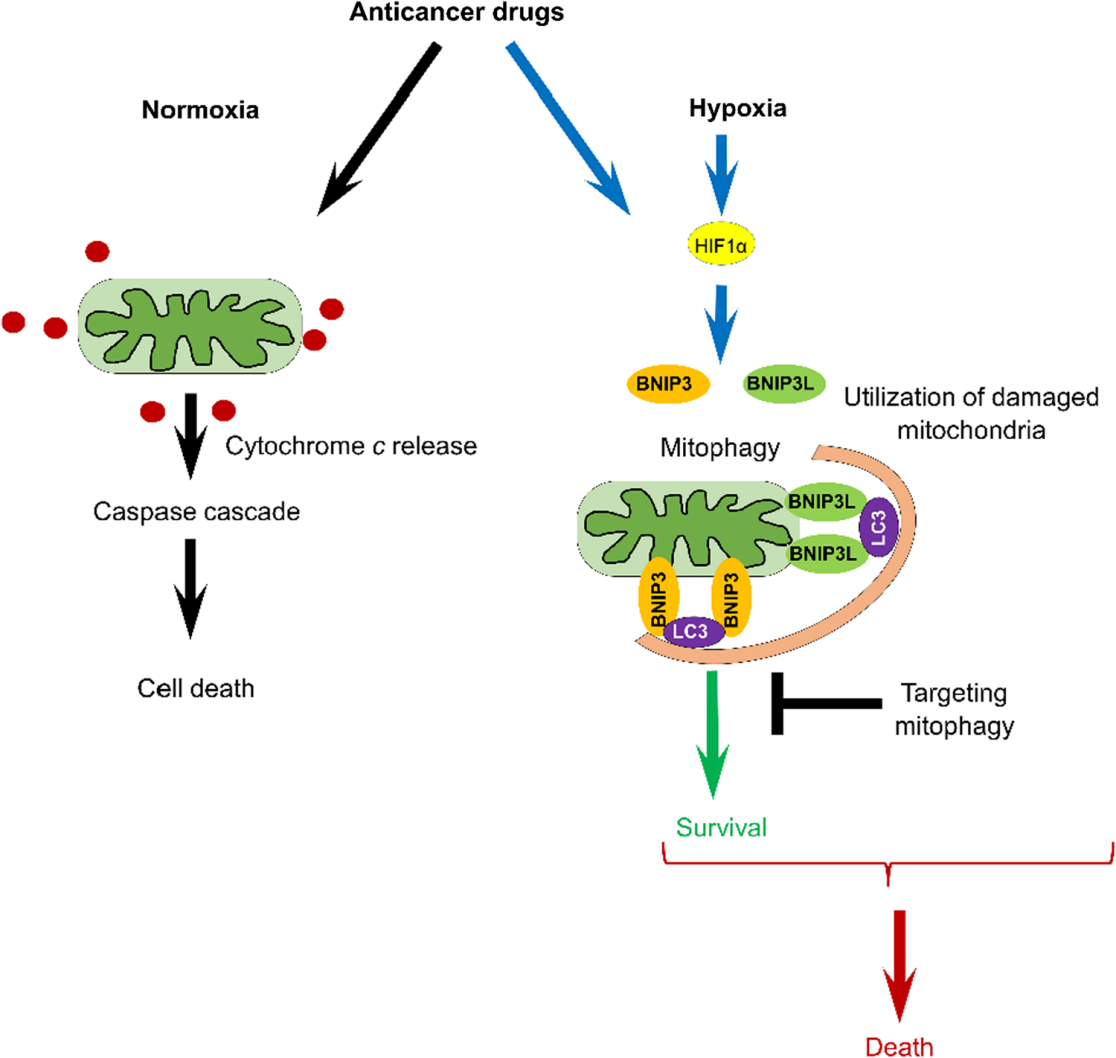
Mitophagy modulates anticancer therapy (see the text for the details)

As we previously discussed, mitophagy plays a dual role in cancer progression, depending on the molecular context and cancer type (Fig. 2) (reviewed in [79]).

Chemotherapeutic agents usually induce mitochondrial dysfunction, accompanied by increased ROS generation and mitophagy in order to exacerbate cytotoxic effects on cancer cells [80]. Excessive mitophagy induction may result in the loss of functional mitochondria, leading to cell death. For instance, dihydroergotamine tartrate (DHE) is a drug for migraine treatment derived from ergot alkaloids (Table 1).

**Table 1:** Drugs that influence the role of mitophagy in various tumors

Drug	Origin	Mechanism of action	Types of cancer
Dihydroergotamine tartrate (DHE)	Ergot alkaloid derivative	PINK1/Parkin-mediated mitophagy, increased ROS production, apoptosis induction	Lung cancer
Ketonazole	Antifungal drug	PINK1/Parkin-mediated mitophagy, COX-2 downregulation, apoptosis induction	HCC
Sorafenib	Multi-kinase inhibitor	PINK1/Parkin-mediated mitophagy, ETC complexes II and III inhibition, apoptosis induction	Renal cancer, liver cancer
AT101	BH3-mimetic	Mitophagic cell death	Glioma cells
Liensinine	Inhibitor of autophagy and mitophagy	Increases the sensitivity of cancer cells to chemotherapeutic drugs	Breast cancer
Melatonin	Hormone	Downregulation of JNK kinase and Parkin, apoptosis induction	Cervical cancer
Mdivi-1	Inhibitor of mitophagy	Enhanced the efficacy of chemotherapeutic drugs	Hepatic carcinoma

It has been demonstrated that DHE induces lung cancer cell death via mitophagy and apoptosis [17]. Specifically, DHE has been shown to induce mitochondrial dysfunction, leading to PINK1/PARKIN-dependent mitophagy activation associated with increased ROS production and further apoptosis in lung cancer cells [17]. In another study, it was shown that the antifungal drug ketoconazole induces apoptosis by triggering PINK1/Parkin-mediated mitophagy and by downregulating COX-2 in HCC [81]. Likewise, the multi-kinase inhibitor sorafenib stimulates apoptosis in renal and liver cancer cells through PINK1/Parkin-mediated mitophagy [82]. Sorafenib has been reported to inhibit complexes II and III of the electron transport chain by stabilizing PINK1 on the OMM and Parkin recruitment to damaged mitochondria, thereby initiating mitophagy [85]. Mitophagy-related cell death can be activated by the induction of ceramide stress in different cancers [83]. Thus, ceramide CerS1 overproduction causes mitophagy and caspase-independent cell death [84]. Additionally, a novel BH3-mimetic AT101 and sodium selenite induced excessive mitophagic cell death in glioma cells [85, 86]. On the other hand, several drugs have been shown to provide antitumor effects by inhibiting mitophagy. For instance, the novel inhibitor of autophagy and mitophagy liensinine increases the sensitivity of breast cancer cells to chemotherapeutic agents that induce mitochondrial fission [87].

The inhibition of mitophagy may also contribute to drug resistance modulation in cancer cells [88]. In cervical cancer, treatment with melatonin, an endogenous indoleamine and antioxidant, has been shown to suppress resistance to cisplatin, thereby restoring the efficacy of chemotherapy [88]. In this study, melatonin was found to hinder mitophagy by downregulating c-Jun N-terminal kinase (JNK) and Parkin, leading to cervical cancer cell apoptosis [88]. Similarly, in hepatic carcinoma, treatment with the inhibitor of Drp1-mediated mitophagy Mdivi-1 or the lysosome inhibitor Bafilomycin A enhanced the efficacy of chemotherapeutic drugs such as cisplatin [89]. Therefore, targeting mitophagy may significantly increase the efficacy of various chemotherapeutic agents.

Mitophagy may contribute to cancer cell survival by adapting to stress conditions, but it may also lead to cell death via excessive mitochondrial clearance (Fig. 2). Therefore, the modulation of mitophagy may represent a novel promising approach for anti-cancer therapies.

## Conclusion

Mitophagy plays important role in maintaining cell and tissue homeostasis by preventing the accumulation of dysfunctional mitochondria that lead to increased ROS production and cell damage. Recent evidence has demonstrated that mitophagy is involved in the regulation of tumorigenesis and tumor progression. Mitophagy modulation seems to be a promising approach for cancer treatment. However, in tumors mitophagy appeared to play dual role in cancer progression. On the one hand, mitophagy inhibits tumor progression by limiting ROS production, while on the other hand, mitophagy may promote tumor growth providing adaptation of tumor cells to the changing microenvironment. Excessive mitophagy in cancer cells may lead to the mitophagic cell death, and at the same time in several tumors the inhibition of mitophagy resulted in suppression of tumor growth. Despite recent advances in understanding of mitophagy mechanisms the role of mitophagy in tumorigenesis appears to be very complex, depending on the type and stage of the tumor. Here we highlighted the most important knowledge regarding mitophagy mechanisms and their role in cancer progression and therapy. However, there are still many questions to be answered. Therefore, further studies are required for better understand the molecular mechanism and function of mitophagy for the development of novel approaches to cancer treatment.
